# Supplementary material for: Obstructive sleep apnea and outcomes in acute pulmonary embolism: A large-scale database study
Source: PLoS One. 2026 Mar 13;21(3):e0342850. doi: 10.1371/journal.pone.0342850 (PMC12987454; doi:10.1371/journal.pone.0342850)
Supplement: S1 File — Supplement table: Codes. (DOCX) [file pone.0342850.s001.docx]

**Figure 1:** **Propensity Score Matching for OSA and without OSA Groups with Acute PE**


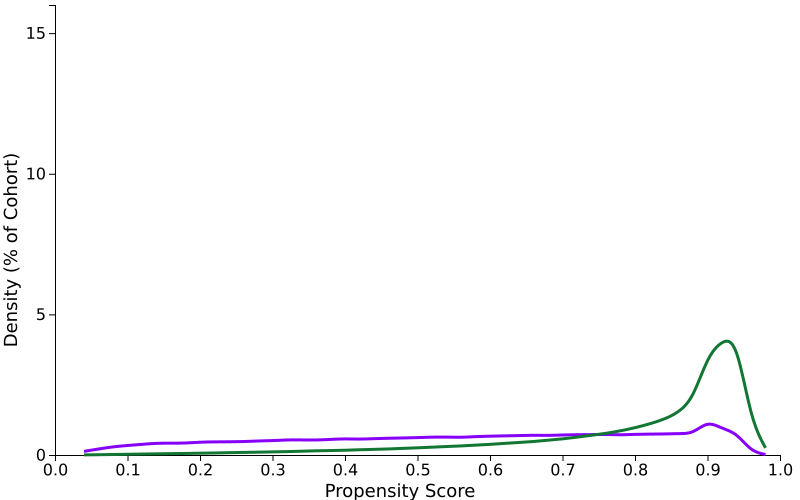

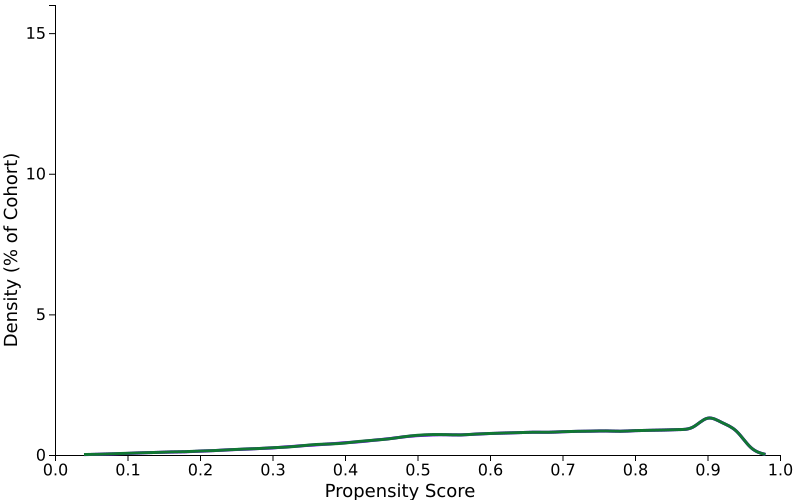


**Abbreviations:** PE = pulmonary embolism; OSA = obstructive sleep apnea

**Supplemental table: codes**

| **Demographics** | |
| --- | --- |
| AI | Age at Index |
| M | Male |
| F | Female |
| 2186-5 | Not Hispanic or Latino |
| **Diagnosis** | |
| C00-D49 | Neoplasms |
| I00-I99 | Diseases of the circulatory system |
| J00-J99 | Diseases of the respiratory system |
| E00-E89 | Endocrine, nutritional and metabolic diseases |
| G89-G99 | Other disorders of the nervous system |
| K00-K95 | Diseases of the digestive system |
| F01-F99 | Mental, behavioral and neurodevelopmental disorders |
| D50-D89 | Diseases of the blood and blood-forming organs and certain disorders involving the immune mechanism |
| M00-M99 | Diseases of the musculoskeletal system and connective tissue |
| **Medication** | |
| BL110 | Anticoagulant |
| BL117 | Platelet aggregation inhibitors |
| 1191 | aspirin |
| **Laboratory** | |
| 9083 | BMI |

| **Cardiac arrest** | |
| --- | --- |
| ICD10CM:I46 | Cardiac arrest |
| ICD10CM:I46.9 | Cardiac arrest, cause unspecified |
| **Mortality** | |
| Deceased | Deceased |
| **Critical care services** | |
| CPT:1013729 | Critical care services |
| **Intubation** | |
| CPT:31500 | Intubation, endotracheal, emergency procedure |
| **Ventilation assist** | |
| ICD10PCS:5A1935Z | **Respiratory ventilation, less than 24 consecutive hours** |
| ICD10PCS:5A1945Z | Respiratory ventilation, 24-96 consecutive hours |
| ICD10PCS:5A1955Z | Respiratory ventilation, greater than 96 consecutive hours |
| **GI bleed** | |
| ICD10CM:K92 | Other diseases of digestive system |
| **Subdural hemorrhage** | |
| ICD10CM:I62.0 | Nontraumatic subdural hemorrhage |
