# Supplementary material for: Obstructive sleep apnea and outcomes in acute pulmonary embolism: A large-scale database study
Source: PLoS One. 2026 Mar 13;21(3):e0342850. doi: 10.1371/journal.pone.0342850 (PMC12987454; doi:10.1371/journal.pone.0342850)
Supplement: S2 File — (DOCX) [file pone.0342850.s002.docx]

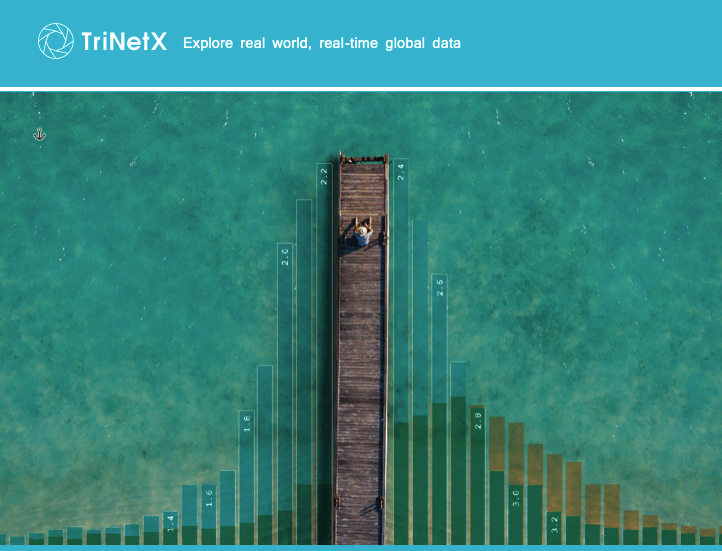


OSA and PE

Compare Outcomes Analysis

Created by TriNetX on Aug 16, 2025, 17:27:41 UTC

# Introduction

TriNetX is the global federated health research network providing access to electronic medical records (diagnoses, procedures, medications, laboratory values, genomic information) across large healthcare organizations (HCOs). This report was run on the set of HCOs grouped into a network called US Collaborative Network. This network included 71 HCO(s).

This report describes a Compare Outcomes Analysis, named PE in OSA vs population without sleep disorder V4 (1), generated by the TriNetX platform on Aug 16, 2025, 17:27:41 UTC. This analysis compared the outcomes of two cohorts: Cohort A (142,759 patients) named OSA + PE V4 and Cohort B (530,683 patients) named PE population w/o sleep disorder V4.

This analysis was run by Saud Alawad (saud.alawad@hsc.wvu.edu) and downloaded by Saud Alawad (saud.alawad@hsc.wvu.edu).

# Methods

The analysis process includes two main steps: 1) Defining the cohorts through query criteria; 2) Setting up and running the analysis. Setting up the analysis requires definitions for the index event, outcomes criteria, and the time frame. Compare outcomes supports four analyses: Measures of Association, Survival, Number of Instances and Lab result distribution. These analyses have additional options that are listed in the Outcomes Definitions and Analyses Specifications section below. Furthermore, characteristics of the cohorts that are balanced using propensity score matching are also included in the Propensity Score Matching section.

## Cohorts definition

This section lists all terms used in the definitions of the two cohorts.

### Query Criteria for Cohort 1 (query name: OSA + PE V4)

This query was run on the network US Collaborative Network with 71 HCO(s) queried and 71 HCO(s) responded. A total of 70 provider(s) responded with patients. The final cohort included 142,759 patients who matched the query criteria listed in the table below. For the text representation of the query criteria please see Appendix A.

|  | | | | | |
| --- | --- | --- | --- | --- | --- |
| Ungrouped terms | | | | | |
|  | must have |  | demographics | Age | Age (at least 18 years (most recent occurrence)) |
| Group 1 | | | | | |
|  | **Group 1A** | | | | |
|  | must have |  | diagnosis | UMLS:ICD10CM:G47.33 | Obstructive sleep apnea (adult) (pediatric) |
|  | date constraint | | The terms in this group occurred between Jan 1, 2013 and Jun 30, 2025 | | |
|  | event relationship | | Any instance of Group 1B occurred on or after any instance of Group 1A | | |
|  | **Group 1B** | | | | |
|  | must have |  | diagnosis | UMLS:ICD10CM:I26 | Pulmonary embolism |

### Query Criteria for Cohort 2 (query name: PE population w/o sleep disorder V4)

This query was run on the network US Collaborative Network with 71 HCO(s) queried and 71 HCO(s) responded. A total of 71 provider(s) responded with patients. The final cohort included 530,683 patients who matched the query criteria listed in the table below.

| Ungrouped terms | | | | | |
| --- | --- | --- | --- | --- | --- |
|  | must have |  | demographics | Age | Age (at least 18 years (most recent occurrence)) |
| Group 1 | | | | | |
|  | **Group 1A** | | | | |
|  | must have |  | diagnosis | UMLS:ICD10CM:I26 | Pulmonary embolism |
|  | cannot have |  | diagnosis | UMLS:ICD10CM:G47 | Sleep disorders |
|  | date constraint | | The terms in this group occurred between Jan 1, 2013 and Jun 30, 2025 | | |

## Analysis Setup

This section contains the Index Event and Time Window definitions and a list of selected outcomes and the analyses.

### Index Event & Time Window Definitions

The index event defines the point in time when each patient in the cohort enters the analysis. To define an index event for the cohort, one or more criteria for the cohort must be selected. The index date for each patient within a cohort is the day on which the patient first met the selected criteria for the cohort (listed in the table below).

As the index event defines the earliest time point after which outcomes are analyzed, the time window defines the duration during which outcomes are analyzed. The time window can start on the same day as the index event or at any specified time interval after the index event. The time window can end any time after the start date. Outcomes are defined as diagnoses, medications, procedures, or laboratory values that happened in the time window starting after the first occurrence of the index event.

### Time Window Used in this Analysis

This analysis included outcomes that occurred in the time window that started 1 day after the first occurrence of the index event and ended 30 days after the first occurrence of the index event.

The index event only includes events that occurred up to 20 years ago. Patients whose index event occurred 20 years or more ago are excluded. In this analysis, 0 patients in Cohort 1 and 0 patients in Cohort 2 were excluded because they met the index event more than 20 years ago.

### Index Events Used in this Analysis

Index events for the Compare Outcomes analysis were derived from the cohort definitions. Index events were defined separately for each cohort and were based on the criteria used in the original cohort definition. Please see Appendix B for the text representation of the index event definition.

The index event for Cohort 1 (query name: OSA + PE V4) was defined as the following:

|  | | | | | |
| --- | --- | --- | --- | --- | --- |
| Group 1 | | | | | |
|  | **Group 1A** | | | | |
|  | must have |  | diagnosis | UMLS:ICD10CM:G47.33 | Obstructive sleep apnea (adult) (pediatric) |
|  | date constraint | | The terms in this group occurred between Jan 1, 2013 and Jun 30, 2025 | | |
|  | event relationship | | Any instance of Group 1B occurred on or after any instance of Group 1A | | |
|  | **Group 1B** | | | | |
|  | must have |  | diagnosis | UMLS:ICD10CM:I26 | Pulmonary embolism |

The index event for Cohort 2 (query name: PE population w/o sleep disorder V4) was defined as the following:

|  | | | | | |
| --- | --- | --- | --- | --- | --- |
| Group 1 | | | | | |
|  | **Group 1A** | | | | |
|  | must have |  | diagnosis | UMLS:ICD10CM:I26 | Pulmonary embolism |
|  | cannot have |  | diagnosis | UMLS:ICD10CM:G47 | Sleep disorders |
|  | date constraint | | The terms in this group occurred between Jan 1, 2013 and Jun 30, 2025 | | |

### Analyses Specifications

The Compare Outcomes Analytic supports four types of analyses: Measure of Association, Survival, Number of Instances, and Lab result distribution. The first three analyses support the “exclude patients with outcomes prior to the window” setting. This option can exclude patients from the analysis if they are not at risk for an outcome (e.g., if the outcome is a chronic disease). When "exclude patients with the outcome prior to the time window" is not checked, all patients in the cohort are included in the analysis, regardless of whether they had the outcome prior to the time window. When "exclude patients with the outcome prior to the time window" is checked, patients are excluded from the analysis if their record includes the outcome prior to the beginning of the time window. This selection will exclude all patients with the outcome prior to the index event. If the start of the time window for the analysis falls some days after the index event, patients will also be excluded if they have the outcome between the index event and the start of the time window.

### Measure of Association Analysis

The Measure of Association Analysis calculates and compares the fraction of patients with the selected outcome. The output summary includes: Patients in each Cohort (count of patients meeting query criteria); Patients with Outcome in each Cohort (of the patients in the cohort, count of patients that had the outcome in the time window); and Risk (the fraction of patients in the cohort that have the outcome in the time window, i.e. Patients with Outcome / Patients in Cohort). In addition, Risk Difference (the difference in the risks in Cohort 1 and Cohort 2), Risk Ratio (the ratio of the risks in Cohort 1 and Cohort 2), and Odds Ratio (the ratio of the odds in Cohort 1 and Cohort 2). The bar chart shows the risk of the outcome for the both cohorts.

### Survival Analysis

The Kaplan-Meier Analysis estimates probability of the outcome at a respective time interval (daily time interval is used in this analysis). In order to account for the patients who exited the cohort during the analysis period, and therefore should not be included in the analysis, censoring is applied. In this analysis, patients are removed from the analysis (censored) after the last fact in their record.

The output summary includes: Patients in each Cohort (count of patients meeting query criteria); Patients with Outcome (of the patients in the cohort, count of patients that had the outcome in the time window); Median Survival (the number of days when the survival drops below 50%; the “-” indicates that survival does not drop below 50% during the time window); and Survival Probability at End of Time Window (the % survival at the end of the time window). In addition, Log-Rank test, Hazard Ratio and test for Proportionality.

### Number of Instances Analysis

The Number of Instances Analysis calculates how many times the outcome occurred in the time window. This analysis includes two additional settings: include patients with zero instances; the definition of an instance.

Selecting to exclude patients with zero instances will remove these patients from the calculations for mean number of instances, standard deviation, or median. The histogram showing the distribution of patients by number of instances will not contain a bar for zero. Alternatively, by selecting to include patients with zero instances, the mean, standard deviation, and median for number of instances will reflect these patients. The histogram will contain a bar for zero patients.

The definition of an instance affects how counts are analyzed. By selecting Date, each calendar date on which any of the terms selected in the outcome are recorded will represent one instance. For example, if the outcome is “Med A or Med B,” and a patient has “Med A” on January 3, then both medications on January 4, then “Med B” on January 6, then that patient is considered to have three instances– January 3, January 4, and January 6. Note that if an outcome occurs across several dates (e.g. Visit: inpatient encounter), then only the start date is tracked for the purpose of counting instances. A patient who begins at stay on January 1, ends that stay on January 3, begins another stay on January 10, and ends that stay on January 15, is considered to have two instances of the outcome.

Selecting Visit as an Instance will count any visit that includes the outcome as one instance, regardless of how many times it occurred. For instance, consider a patient administered an analgesic on each of the three days that make up an inpatient stay following some index event. If analgesic is an outcome, these three administrations will represent only one instance, because all three are associated with the same visit.

The output summary includes: Patients in Cohort (count of patients meeting query criteria); Patients with Outcome (of the patients in the cohort, count of patients that had the outcome in the time window); Mean (mean of the counts); Standard Deviation (standard deviation of the counts); Median (median of the counts); and Median (1+ instances) when patients with zero instances included in the analysis. In addition, T-Test statistics testing for the difference between the cohorts is included.

### Laboratory Results Analysis

Lab Results can be included in the analysis only for the outcomes that are labs. Only the most recent lab values in the time window are included. For the lab results that are numeric, the outcome summary includes: Patients in Cohort (count of patients meeting query criteria); Patients with Outcome (of the patients in the cohort, count of patients that had the outcome in the time window); Mean (mean of the counts); and Standard Deviation (the standard deviation for lab values across patients in the cohort). In addition, T-Test statistics testing for the difference between the cohorts is included.

For the non-numeric lab results, three values are reported: counts of Negative; Positives; and Unknowns.

The counts are represented in the bar chart as percentages of the total counts.

### Outcome Definitions

Table below outlines the definitions for each outcome and the analysis specifications. For outcome definitions consisting of more than one term, at least one term must match. Please see Appendix C for the text representation of the outcome definitions.

| Mortality | | | | |
| --- | --- | --- | --- | --- |
|  | **Outcome definition** | | | |
|  | | Demographics | Deceased | Deceased |
|  | **Settings for the performed analyses** | | | |
|  | | Kaplan - Meier survival analysis | | excluding patients with outcome prior to the time window |
|  | | Risk analysis | | excluding patients with outcome prior to the time window |
| Cardiac arrest | | | | |
|  | **Outcome definition** | | | |
|  | | Diagnosis | UMLS:ICD10CM:I46 | Cardiac arrest |
|  | | Diagnosis | UMLS:ICD10CM:I46.9 | Cardiac arrest, cause unspecified |
|  | **Settings for the performed analyses** | | | |
|  | | Risk analysis | | excluding patients with outcome prior to the time window |
|  | | Kaplan - Meier survival analysis | | excluding patients with outcome prior to the time window |
| Critical Care services | | | | |
|  | **Outcome definition** | | | |
|  | | Procedure | UMLS:CPT:1013729 | Critical Care Services |
|  | **Settings for the performed analyses** | | | |
|  | | Risk analysis | | excluding patients with outcome prior to the time window |
| Intubation | | | | |
|  | **Outcome definition** | | | |
|  | | Procedure | UMLS:CPT:31500 | Intubation, endotracheal, emergency procedure |
|  | **Settings for the performed analyses** | | | |
|  | | Risk analysis | | excluding patients with outcome prior to the time window |
| Ventilation assist | | | | |
|  | **Outcome definition** | | | |
|  | | Procedure | UMLS:ICD10PCS:5A1935Z | Respiratory Ventilation, Less than 24 Consecutive Hours |
|  | | Procedure | UMLS:ICD10PCS:5A1945Z | Respiratory Ventilation, 24-96 Consecutive Hours |
|  | | Procedure | UMLS:ICD10PCS:5A1955Z | Respiratory Ventilation, Greater than 96 Consecutive Hours |
|  | **Settings for the performed analyses** | | | |
|  | | Risk analysis | | excluding patients with outcome prior to the time window |
| GI bleed | | | | |
|  | **Outcome definition** | | | |
|  | | Diagnosis | UMLS:ICD10CM:K92 | Other diseases of digestive system |
|  | **Settings for the performed analyses** | | | |
|  | | Risk analysis | | excluding patients with outcome prior to the time window |
| Intracerebral bleed | | | | |
|  | **Outcome definition** | | | |
|  | | Diagnosis | UMLS:ICD10CM:I61 | Nontraumatic intracerebral hemorrhage |
|  | **Settings for the performed analyses** | | | |
|  | | Risk analysis | | excluding patients with outcome prior to the time window |
| SDH | | | | |
|  | **Outcome definition** | | | |
|  | | Diagnosis | UMLS:ICD10CM:I62.0 | Nontraumatic subdural hemorrhage |
|  | **Settings for the performed analyses** | | | |
|  | | Risk analysis | | excluding patients with outcome prior to the time window |
| ED | | | | |
|  | **Outcome definition** | | | |
|  | | Medication | NLM:VA:BL115 | THROMBOLYTICS |
|  | **Settings for the performed analyses** | | | |
|  | | Risk analysis | | excluding patients with outcome prior to the time window |
| Unnamed Outcome | | | | |
|  | **Outcome definition** | | | |
|  | | Procedure | UMLS:CPT:1006787 | Venous Mechanical Thrombectomy Procedures |
|  | | Procedure | UMLS:CPT:1006784 | Primary percutaneous transluminal mechanical thrombectomy, noncoronary, arterial or arterial bypass graft, including fluoroscopic guidance and intraprocedural pharmacological thrombolytic injection(s) (deprecated 2018) |
|  | | Procedure | UMLS:CPT:1027831 | Primary percutaneous transluminal mechanical thrombectomy, noncoronary, non-intracranial, arterial or arterial bypass graft, including fluoroscopic guidance and intraprocedural pharmacological thrombolytic injection(s) |
|  | | Procedure | UMLS:CPT:37186 | Secondary percutaneous transluminal thrombectomy (eg, nonprimary mechanical, snare basket, suction technique), noncoronary, non-intracranial, arterial or arterial bypass graft, including fluoroscopic guidance and intraprocedural pharmacological thrombolytic injections, provided in conjunction with another percutaneous intervention other than primary mechanical thrombectomy (List separately in addition to code for primary procedure) |
|  | **Settings for the performed analyses** | | | |
|  | | Risk analysis | | excluding patients with outcome prior to the time window |

## Propensity Score Matching

Propensity score matching was performed on all listed characteristics. Characteristics of the cohorts before and after matching are summarized in the table below.

| **Cohort 1 and cohort 2 patient count before and after propensity score matching** | | | | | | | | | | | | |
| --- | --- | --- | --- | --- | --- | --- | --- | --- | --- | --- | --- | --- |
|  | | | Cohort | | | Patient count before matching | | | | Patient count after matching | | |
|  | | | 1 - OSA + PE V4 | | | 92,461 | | | | 76,636 | | |
|  | | | 2 - PE population w/o sleep disorder V4 | | | 332,141 | | | | 76,636 | | |
| **Propensity score density function - Before and after matching (cohort 1 - purple, cohort 2 - green)** | | | | | | | | | | | | |
|  |  | | 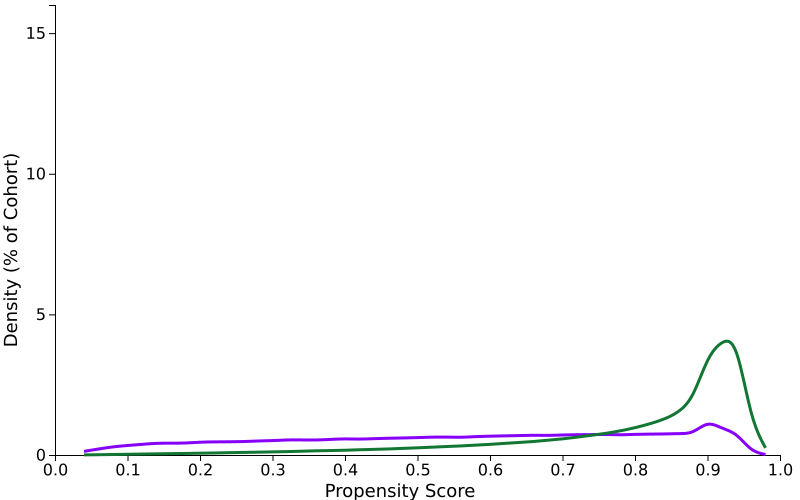 | | | | 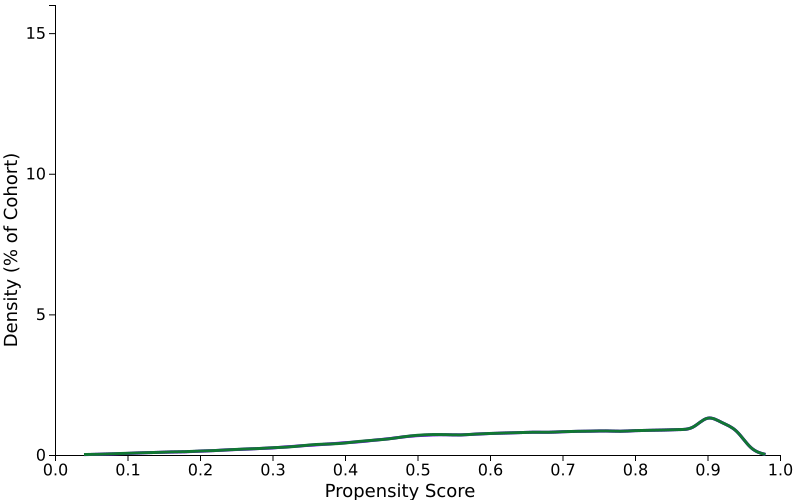 | | | | | |
| **Cohort 1 (N = 92,461) and cohort 2 (N = 332,141) characteristics before propensity score matching** | | | | | | | | | | | | |
|  | **Demographics** | | | | | | | | | | | |
|  |  | Cohort | | |  | Mean ± SD | | Patients | % of Cohort | | P-Value | Std diff. |
|  |  | 1 2 | | AI | Age at Index | 62.6 +/- 13.9 60.6 +/- 17.9 | | 92,461 332,141 | 100% 100% | | <0.001 | 0.124 |
|  |  | 1 2 | | 2106-3 | White |  | | 69,056 230,713 | 74.7% 69.5% | | <0.001 | 0.117 |
|  |  | 1 2 | | F | Female |  | | 43,013 175,914 | 46.5% 53.0% | | <0.001 | 0.129 |
|  |  | 1 2 | | UN | Unknown Ethnicity |  | | 13,547 55,826 | 14.7% 16.8% | | <0.001 | 0.059 |
|  |  | 1 2 | | 2054-5 | Black or African American |  | | 15,805 62,044 | 17.1% 18.7% | | <0.001 | 0.041 |
|  |  | 1 2 | | 2186-5 | Not Hispanic or Latino |  | | 74,509 256,191 | 80.6% 77.1% | | <0.001 | 0.085 |
|  |  | 1 2 | | M | Male |  | | 49,430 155,905 | 53.5% 46.9% | | <0.001 | 0.131 |
|  | **Diagnosis** | | | | | | | | | | | |
|  |  | Cohort | | |  | Mean ± SD | | Patients | % of Cohort | | P-Value | Std diff. |
|  |  | 1 2 | | K00-K95 | Diseases of the digestive system |  | | 62,840 124,624 | 68.0% 37.5% | | <0.001 | 0.640 |
|  |  | 1 2 | | C00-D49 | Neoplasms |  | | 38,194 92,825 | 41.3% 27.9% | | <0.001 | 0.284 |
|  |  | 1 2 | | D50-D89 | Diseases of the blood and blood-forming organs and certain disorders involving the immune mechanism |  | | 46,089 89,067 | 49.8% 26.8% | | <0.001 | 0.488 |
|  |  | 1 2 | | I10-I1A | Hypertensive diseases |  | | 65,447 117,903 | 70.8% 35.5% | | <0.001 | 0.756 |
|  |  | 1 2 | | I20-I25 | Ischemic heart diseases |  | | 30,882 44,539 | 33.4% 13.4% | | <0.001 | 0.486 |
|  |  | 1 2 | | I30-I5A | Other forms of heart disease |  | | 51,356 84,440 | 55.5% 25.4% | | <0.001 | 0.645 |
|  |  | 1 2 | | I80-I89 | Diseases of veins, lymphatic vessels and lymph nodes, not elsewhere classified |  | | 30,157 45,825 | 32.6% 13.8% | | <0.001 | 0.457 |
|  |  | 1 2 | | I70-I79 | Diseases of arteries, arterioles and capillaries |  | | 22,336 34,135 | 24.2% 10.3% | | <0.001 | 0.374 |
|  |  | 1 2 | | E08-E13 | Diabetes mellitus |  | | 34,381 46,475 | 37.2% 14.0% | | <0.001 | 0.551 |
|  |  | 1 2 | | K70-K77 | Diseases of liver |  | | 14,928 23,682 | 16.1% 7.1% | | <0.001 | 0.284 |
|  |  | 1 2 | | J40-J4A | Chronic lower respiratory diseases |  | | 37,815 50,851 | 40.9% 15.3% | | <0.001 | 0.594 |
|  |  | 1 2 | | F17 | Nicotine dependence |  | | 15,229 34,151 | 16.5% 10.3% | | <0.001 | 0.183 |
|  |  | 1 2 | | F10 | Alcohol related disorders |  | | 4,937 11,253 | 5.3% 3.4% | | <0.001 | 0.096 |
|  | **Medication** | | | | | | | | | | | |
|  |  | Cohort | | |  | Mean ± SD | | Patients | % of Cohort | | P-Value | Std diff. |
|  |  | 1 2 | | BL110 | ANTICOAGULANTS |  | | 60,318 124,539 | 65.2% 37.5% | | <0.001 | 0.578 |
|  |  | 1 2 | | B01AC | Platelet aggregation inhibitors excl. heparin |  | | 38,996 68,186 | 42.2% 20.5% | | <0.001 | 0.480 |
|  |  | 1 2 | | 1191 | aspirin |  | | 37,374 64,803 | 40.4% 19.5% | | <0.001 | 0.469 |
|  | **Laboratory** | | | | | | | | | | | |
|  |  | Cohort | | |  | Mean ± SD | | Patients | % of Cohort | | P-Value | Std diff. |
|  |  | 1 2 | | 9083 | BMI | 36.1 +/- 9.6 29.1 +/- 7.3 | | 60,710 154,901 | 65.7% 46.6% | | <0.001 | 0.820 |
|  |  | 1 2 | |  | 0 - 18.50 kg/m2 |  | | 2,394 10,922 | 2.6% 3.3% | | <0.001 | 0.041 |
|  |  | 1 2 | |  | 18.50 - 24.90 kg/m2 |  | | 9,404 56,265 | 10.2% 16.9% | | <0.001 | 0.199 |
|  |  | 1 2 | |  | 25 - 29.90 kg/m2 |  | | 20,986 75,819 | 22.7% 22.8% | | 0.404 | 0.003 |
|  |  | 1 2 | |  | 30 - 34.90 kg/m2 |  | | 27,343 55,473 | 29.6% 16.7% | | <0.001 | 0.309 |
|  |  | 1 2 | |  | 35 - 39.90 kg/m2 |  | | 24,692 29,134 | 26.7% 8.8% | | <0.001 | 0.483 |
|  |  | 1 2 | |  | 40 - 0 kg/m2 |  | | 25,232 18,766 | 27.3% 5.7% | | <0.001 | 0.610 |
|  |  | 1 2 | | 9014 | Hemoglobin [Mass/volume] in Blood | 12.6 +/- 2.4 12.3 +/- 2.4 | | 70,984 174,427 | 76.8% 52.5% | | <0.001 | 0.147 |
|  |  | 1 2 | |  | 0 - 0 g/dL |  | | 70,984 174,430 | 76.8% 52.5% | | <0.001 | 0.525 |
|  |  | 1 2 | | 9020 | Platelets [#/volume] in Blood | 242.4 +/- 94.3 251.4 +/- 108.5 | | 70,695 173,853 | 76.5% 52.3% | | <0.001 | 0.089 |
|  |  | 1 2 | |  | 0 - 0 10*3/uL |  | | 70,696 173,859 | 76.5% 52.3% | | <0.001 | 0.520 |
|  |  | 1 2 | | 9032 | INR in Plasma or Blood | 1.4 +/- 0.7 1.3 +/- 0.6 | | 49,916 107,672 | 54.0% 32.4% | | <0.001 | 0.107 |
|  |  | 1 2 | |  | 0 - 0 {INR} |  | | 49,916 107,673 | 54.0% 32.4% | | <0.001 | 0.446 |
|  |  | 1 2 | | 9021 | Bicarbonate [Moles/volume] in Serum, Plasma or Blood | 26.0 +/- 3.9 25.5 +/- 3.6 | | 71,988 176,559 | 77.9% 53.2% | | <0.001 | 0.153 |
|  |  | 1 2 | |  | 0 - 0 mmol/L |  | | 71,988 176,559 | 77.9% 53.2% | | <0.001 | 0.538 |
|  |  | 1 2 | | 9024 | Creatinine [Mass/volume] in Serum, Plasma or Blood | 1.2 +/- 2.1 1.1 +/- 1.7 | | 71,124 176,146 | 76.9% 53.0% | | <0.001 | 0.069 |
|  |  | 1 2 | |  | 0 - 0 mg/dL |  | | 71,124 176,146 | 76.9% 53.0% | | <0.001 | 0.517 |
| **Cohort 1 (N = 76,636) and cohort 2 (N = 76,636) characteristics after propensity score matching** | | | | | | | | | | | | |
|  | **Demographics** | | | | | | | | | | | |
|  |  | Cohort | | |  | Mean ± SD | | Patients | % of Cohort | | P-Value | Std diff. |
|  |  | 1 2 | | AI | Age at Index | 62.7 +/- 14.1 63.6 +/- 15.9 | | 76,636 76,636 | 100% 100% | | <0.001 | 0.058 |
|  |  | 1 2 | | 2106-3 | White |  | | 56,579 57,006 | 73.8% 74.4% | | 0.013 | 0.013 |
|  |  | 1 2 | | F | Female |  | | 35,992 35,586 | 47.0% 46.4% | | 0.038 | 0.011 |
|  |  | 1 2 | | UN | Unknown Ethnicity |  | | 11,377 11,392 | 14.8% 14.9% | | 0.914 | 0.001 |
|  |  | 1 2 | | 2054-5 | Black or African American |  | | 13,454 13,254 | 17.6% 17.3% | | 0.178 | 0.007 |
|  |  | 1 2 | | 2186-5 | Not Hispanic or Latino |  | | 61,516 61,557 | 80.3% 80.3% | | 0.792 | 0.001 |
|  |  | 1 2 | | M | Male |  | | 40,630 40,955 | 53.0% 53.4% | | 0.096 | 0.008 |
|  | **Diagnosis** | | | | | | | | | | | |
|  |  | Cohort | | |  | Mean ± SD | | Patients | % of Cohort | | P-Value | Std diff. |
|  |  | 1 2 | | K00-K95 | Diseases of the digestive system |  | | 48,445 48,512 | 63.2% 63.3% | | 0.723 | 0.002 |
|  |  | 1 2 | | C00-D49 | Neoplasms |  | | 30,126 30,029 | 39.3% 39.2% | | 0.612 | 0.003 |
|  |  | 1 2 | | D50-D89 | Diseases of the blood and blood-forming organs and certain disorders involving the immune mechanism |  | | 34,792 34,606 | 45.4% 45.2% | | 0.340 | 0.005 |
|  |  | 1 2 | | I10-I1A | Hypertensive diseases |  | | 50,324 50,698 | 65.7% 66.2% | | 0.044 | 0.010 |
|  |  | 1 2 | | I20-I25 | Ischemic heart diseases |  | | 22,198 22,241 | 29.0% 29.0% | | 0.809 | 0.001 |
|  |  | 1 2 | | I30-I5A | Other forms of heart disease |  | | 38,013 38,000 | 49.6% 49.6% | | 0.947 | <0.001 |
|  |  | 1 2 | | I80-I89 | Diseases of veins, lymphatic vessels and lymph nodes, not elsewhere classified |  | | 21,186 20,973 | 27.6% 27.4% | | 0.223 | 0.006 |
|  |  | 1 2 | | I70-I79 | Diseases of arteries, arterioles and capillaries |  | | 16,009 16,040 | 20.9% 20.9% | | 0.846 | 0.001 |
|  |  | 1 2 | | E08-E13 | Diabetes mellitus |  | | 23,908 23,766 | 31.2% 31.0% | | 0.433 | 0.004 |
|  |  | 1 2 | | K70-K77 | Diseases of liver |  | | 10,655 10,491 | 13.9% 13.7% | | 0.224 | 0.006 |
|  |  | 1 2 | | J40-J4A | Chronic lower respiratory diseases |  | | 26,296 26,336 | 34.3% 34.4% | | 0.830 | 0.001 |
|  |  | 1 2 | | F17 | Nicotine dependence |  | | 11,708 11,586 | 15.3% 15.1% | | 0.385 | 0.004 |
|  |  | 1 2 | | F10 | Alcohol related disorders |  | | 3,821 3,771 | 5.0% 4.9% | | 0.556 | 0.003 |
|  | **Medication** | | | | | | | | | | | |
|  |  | Cohort | | |  | Mean ± SD | | Patients | % of Cohort | | P-Value | Std diff. |
|  |  | 1 2 | | BL110 | ANTICOAGULANTS |  | | 46,299 46,334 | 60.4% 60.5% | | 0.855 | 0.001 |
|  |  | 1 2 | | B01AC | Platelet aggregation inhibitors excl. heparin |  | | 28,893 29,008 | 37.7% 37.9% | | 0.545 | 0.003 |
|  |  | 1 2 | | 1191 | aspirin |  | | 27,618 27,714 | 36.0% 36.2% | | 0.610 | 0.003 |
|  | **Laboratory** | | | | | | | | | | | |
|  |  | Cohort | | |  | Mean ± SD | | Patients | % of Cohort | | P-Value | Std diff. |
|  |  | 1 2 | | 9083 | BMI | 34.5 +/- 9.3 32.7 +/- 8.1 | | 46,589 46,188 | 60.8% 60.3% | | <0.001 | 0.214 |
|  |  | 1 2 | |  | 0 - 18.50 kg/m2 |  | | 2,032 1,915 | 2.7% 2.5% | | 0.059 | 0.010 |
|  |  | 1 2 | |  | 18.50 - 24.90 kg/m2 |  | | 9,087 8,712 | 11.9% 11.4% | | 0.003 | 0.015 |
|  |  | 1 2 | |  | 25 - 29.90 kg/m2 |  | | 18,998 19,050 | 24.8% 24.9% | | 0.758 | 0.002 |
|  |  | 1 2 | |  | 30 - 34.90 kg/m2 |  | | 21,844 21,808 | 28.5% 28.5% | | 0.839 | 0.001 |
|  |  | 1 2 | |  | 35 - 39.90 kg/m2 |  | | 16,093 15,923 | 21.0% 20.8% | | 0.285 | 0.005 |
|  |  | 1 2 | |  | 40 - 0 kg/m2 |  | | 13,852 13,482 | 18.1% 17.6% | | 0.014 | 0.013 |
|  |  | 1 2 | | 9014 | Hemoglobin [Mass/volume] in Blood | 12.7 +/- 2.3 12.1 +/- 2.4 | | 56,125 56,041 | 73.2% 73.1% | | <0.001 | 0.241 |
|  |  | 1 2 | |  | 0 - 0 g/dL |  | | 56,125 56,041 | 73.2% 73.1% | | 0.628 | 0.002 |
|  |  | 1 2 | | 9020 | Platelets [#/volume] in Blood | 243.4 +/- 94.6 246.1 +/- 107.8 | | 55,891 55,832 | 72.9% 72.9% | | <0.001 | 0.026 |
|  |  | 1 2 | |  | 0 - 0 10*3/uL |  | | 55,891 55,833 | 72.9% 72.9% | | 0.739 | 0.002 |
|  |  | 1 2 | | 9032 | INR in Plasma or Blood | 1.4 +/- 0.7 1.3 +/- 0.6 | | 38,277 38,185 | 49.9% 49.8% | | <0.001 | 0.068 |
|  |  | 1 2 | |  | 0 - 0 {INR} |  | | 38,277 38,185 | 49.9% 49.8% | | 0.638 | 0.002 |
|  |  | 1 2 | | 9021 | Bicarbonate [Moles/volume] in Serum, Plasma or Blood | 25.9 +/- 3.8 25.5 +/- 3.7 | | 56,998 56,972 | 74.4% 74.3% | | <0.001 | 0.107 |
|  |  | 1 2 | |  | 0 - 0 mmol/L |  | | 56,998 56,972 | 74.4% 74.3% | | 0.879 | 0.001 |
|  |  | 1 2 | | 9024 | Creatinine [Mass/volume] in Serum, Plasma or Blood | 1.1 +/- 1.9 1.1 +/- 1.8 | | 56,480 56,416 | 73.7% 73.6% | | 0.942 | <0.001 |
|  |  | 1 2 | |  | 0 - 0 mg/dL |  | | 56,480 56,416 | 73.7% 73.6% | | 0.711 | 0.002 |

# Results

Results are summarized in the tables below. Outcomes analysis was performed on the cohorts after propensity score matching.

| **Follow-up Time (Before Matching)** | | | | | | |
| --- | --- | --- | --- | --- | --- | --- |
|  |  | Cohort | Mean Follow-up (Days) | Standard Deviation | Median Follow-up (Days) | Interquartile Range |
|  |  | OSA + PE V4 | 28.654 | 5.353 | 30 | 0 |
|  |  | PE population w/o sleep disorder V4 | 26.898 | 8.074 | 30 | 0 |
| 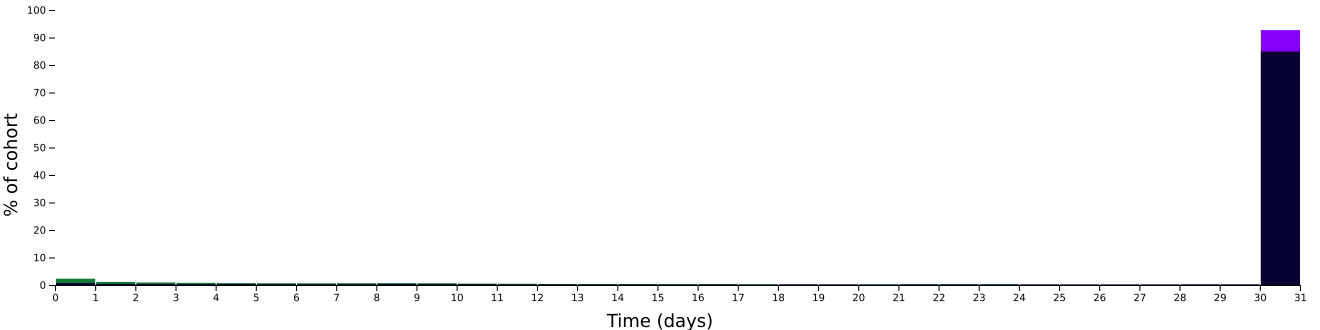 | | | | | | |
|  | | | | | | |
| **Follow-up Time (After Matching)** | | | | | | |
|  |  | Cohort | Mean Follow-up (Days) | Standard Deviation | Median Follow-up (Days) | Interquartile Range |
|  |  | OSA + PE V4 | 28.585 | 5.492 | 30 | 0 |
|  |  | PE population w/o sleep disorder V4 | 27.613 | 7.044 | 30 | 0 |
| 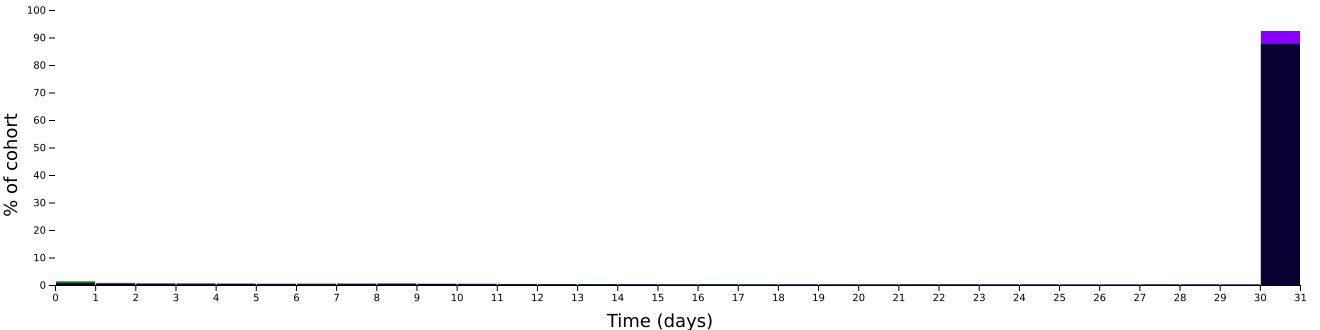 | | | | | | |

| **1 Mortality** | | | | | | | | | | | | |
| --- | --- | --- | --- | --- | --- | --- | --- | --- | --- | --- | --- | --- |
|  | | **Risk analysis excluding patients with outcome prior to the time window** | | | | | | | | | | |
|  |  | | | Cohort | | | Patients in cohort | Patients with outcome | Risk | | | |
|  | | |  | 1 | | OSA + PE V4 | 76,146 | 3,340 | 0.044 | | | |
|  | | |  | 2 | | PE population w/o sleep disorder V4 | 75,705 | 5,979 | 0.079 | | | |
|  | | | | | | | | | | | | |
|  | | |  |  | | |  | 95% CI | z | p |  |  |
|  | | |  | **Risk Difference** | | | -0.035 | (-0.038, -0.033) | -28.506 | 0.000 |  |  |
|  | | |  | **Risk Ratio** | | | 0.555 | (0.533, 0.579) | N/A | N/A |  |  |
|  | | |  | **Odds Ratio** | | | 0.535 | (0.512, 0.559) | N/A | N/A |  |  |
|  | | | | | | | | | | | | |
|  | |  | | | 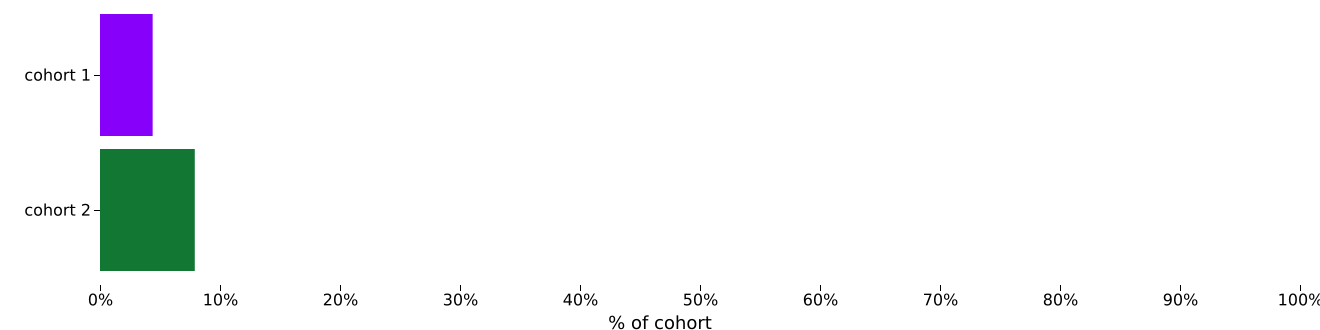 | | | | | | | |
|  | |  | | | 490 patients in Cohort 1 and 931 patients in Cohort 2 were excluded from results because they had the outcome prior to the time window. | | | | | | | |
|  | | **Kaplan - Meier survival analysis excluding patients with outcome prior to the time window** | | | | | | | | | | |
|  | | |  | Cohort | | | Patients in cohort | Patients with outcome | Median survival (days) | Survival probability at end of time window | | |
|  | | |  | 1 | | OSA + PE V4 | 76,146 | 3,340 | -- | 95.51% | | |
|  | | |  | 2 | | PE population w/o sleep disorder V4 | 75,705 | 5,979 | -- | 91.81% | | |
|  | | | | | | | | | | | | |
|  | | |  |  | | | χ^2^ | df | p |  |  |  |
|  | | |  | **Log-Rank Test** | | | 856.355 | 1 | 0.000 |  |  |  |
|  | | | | | | | | | | | | |
|  | | |  |  | | | Hazard Ratio | 95% CI | χ^2^ | df | p | |
|  | | |  | **Hazard Ratio and Proportionality** | | | 0.537 | (0.515, 0.560) | 7.211 | 1 | 0.007 | |
|  | | | | | | | | | | | | |
|  | |  | | | 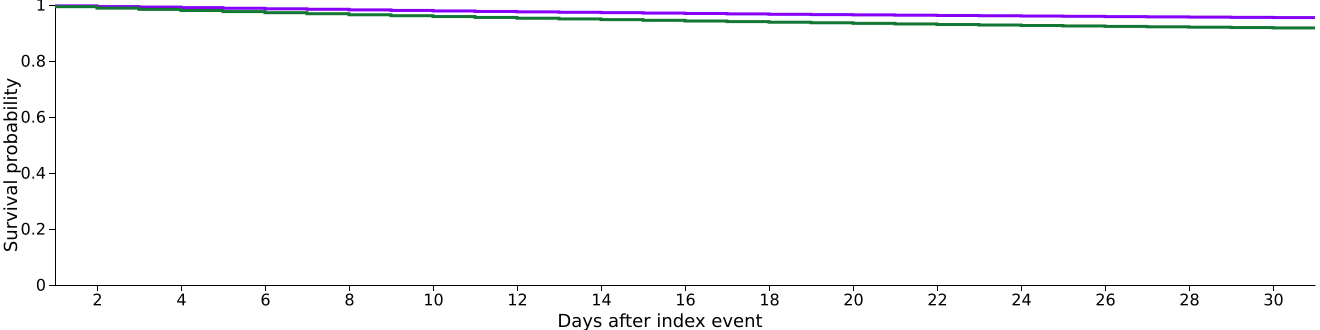 | | | | | | | |
|  | |  | | | 490 patients in Cohort 1 and 931 patients in Cohort 2 were excluded from results because they had the outcome prior to the time window. | | | | | | | |
| **2 Cardiac arrest** | | | | | | | | | | | | |
|  | | **Risk analysis excluding patients with outcome prior to the time window** | | | | | | | | | | |
|  |  | | | Cohort | | | Patients in cohort | Patients with outcome | Risk | | | |
|  | | |  | 1 | | OSA + PE V4 | 74,620 | 228 | 0.003 | | | |
|  | | |  | 2 | | PE population w/o sleep disorder V4 | 74,301 | 357 | 0.005 | | | |
|  | | | | | | | | | | | | |
|  | | |  |  | | |  | 95% CI | z | p |  |  |
|  | | |  | **Risk Difference** | | | -0.002 | (-0.002, -0.001) | -5.396 | 0.000 |  |  |
|  | | |  | **Risk Ratio** | | | 0.636 | (0.539, 0.751) | N/A | N/A |  |  |
|  | | |  | **Odds Ratio** | | | 0.635 | (0.537, 0.750) | N/A | N/A |  |  |
|  | | | | | | | | | | | | |
|  | |  | | | 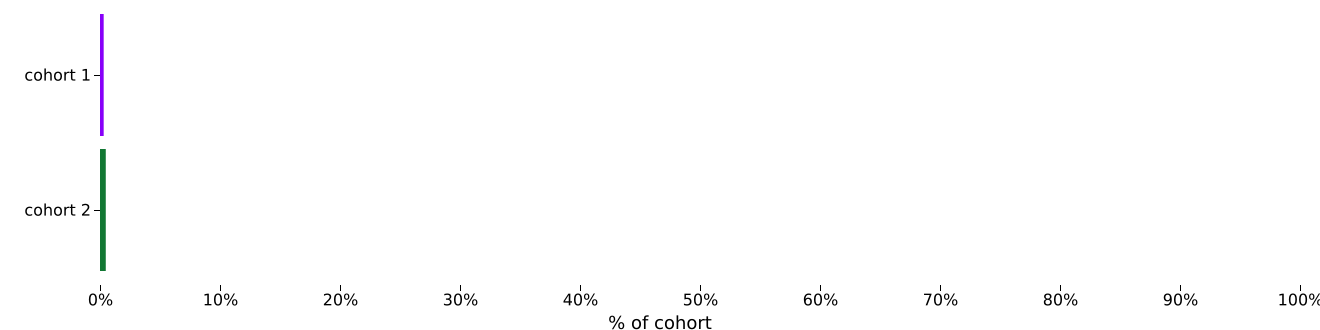 | | | | | | | |
|  | |  | | | 2,016 patients in Cohort 1 and 2,335 patients in Cohort 2 were excluded from results because they had the outcome prior to the time window. | | | | | | | |
|  | | **Kaplan - Meier survival analysis excluding patients with outcome prior to the time window** | | | | | | | | | | |
|  | | |  | Cohort | | | Patients in cohort | Patients with outcome | Median survival (days) | Survival probability at end of time window | | |
|  | | |  | 1 | | OSA + PE V4 | 74,620 | 228 | -- | 99.68% | | |
|  | | |  | 2 | | PE population w/o sleep disorder V4 | 74,301 | 357 | -- | 99.49% | | |
|  | | | | | | | | | | | | |
|  | | |  |  | | | χ^2^ | df | p |  |  |  |
|  | | |  | **Log-Rank Test** | | | 32.388 | 1 | 0.000 |  |  |  |
|  | | | | | | | | | | | | |
|  | | |  |  | | | Hazard Ratio | 95% CI | χ^2^ | df | p | |
|  | | |  | **Hazard Ratio and Proportionality** | | | 0.620 | (0.525, 0.732) | 0.342 | 1 | 0.559 | |
|  | | | | | | | | | | | | |
|  | |  | | | 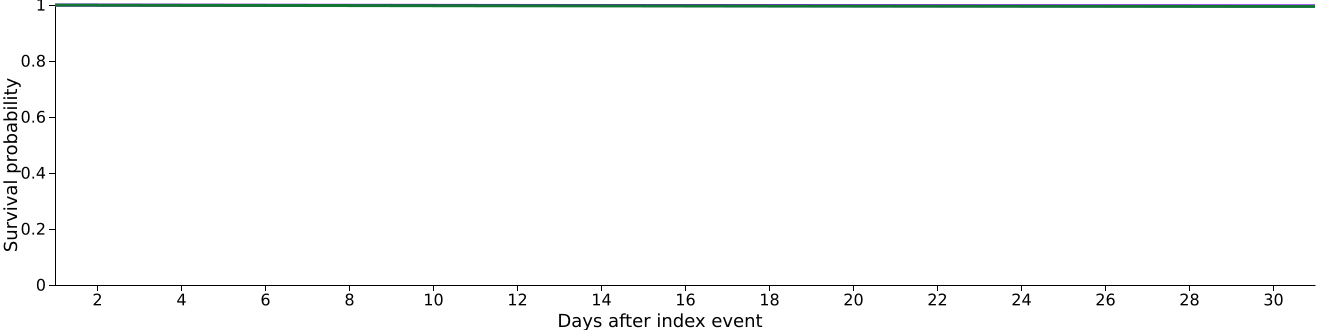 | | | | | | | |
|  | |  | | | 2,016 patients in Cohort 1 and 2,335 patients in Cohort 2 were excluded from results because they had the outcome prior to the time window. | | | | | | | |
| **3 Critical Care services** | | | | | | | | | | | | |
|  | | **Risk analysis excluding patients with outcome prior to the time window** | | | | | | | | | | |
|  |  | | | Cohort | | | Patients in cohort | Patients with outcome | Risk | | | |
|  | | |  | 1 | | OSA + PE V4 | 58,287 | 3,349 | 0.057 | | | |
|  | | |  | 2 | | PE population w/o sleep disorder V4 | 58,495 | 3,488 | 0.060 | | | |
|  | | | | | | | | | | | | |
|  | | |  |  | | |  | 95% CI | z | p |  |  |
|  | | |  | **Risk Difference** | | | -0.002 | (-0.005, 0.001) | -1.581 | 0.114 |  |  |
|  | | |  | **Risk Ratio** | | | 0.964 | (0.920, 1.009) | N/A | N/A |  |  |
|  | | |  | **Odds Ratio** | | | 0.961 | (0.916, 1.010) | N/A | N/A |  |  |
|  | | | | | | | | | | | | |
|  | |  | | | 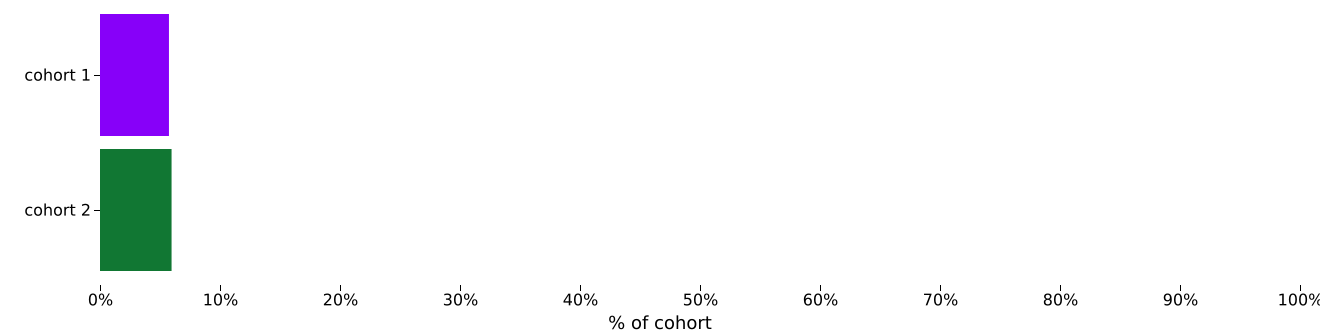 | | | | | | | |
|  | |  | | | 18,349 patients in Cohort 1 and 18,141 patients in Cohort 2 were excluded from results because they had the outcome prior to the time window. | | | | | | | |
| **4 Intubation** | | | | | | | | | | | | |
|  | | **Risk analysis excluding patients with outcome prior to the time window** | | | | | | | | | | |
|  |  | | | Cohort | | | Patients in cohort | Patients with outcome | Risk | | | |
|  | | |  | 1 | | OSA + PE V4 | 73,844 | 1,363 | 0.018 | | | |
|  | | |  | 2 | | PE population w/o sleep disorder V4 | 73,644 | 1,504 | 0.020 | | | |
|  | | | | | | | | | | | | |
|  | | |  |  | | |  | 95% CI | z | p |  |  |
|  | | |  | **Risk Difference** | | | -0.002 | (-0.003, -0.001) | -2.733 | 0.006 |  |  |
|  | | |  | **Risk Ratio** | | | 0.904 | (0.841, 0.972) | N/A | N/A |  |  |
|  | | |  | **Odds Ratio** | | | 0.902 | (0.838, 0.971) | N/A | N/A |  |  |
|  | | | | | | | | | | | | |
|  | |  | | | 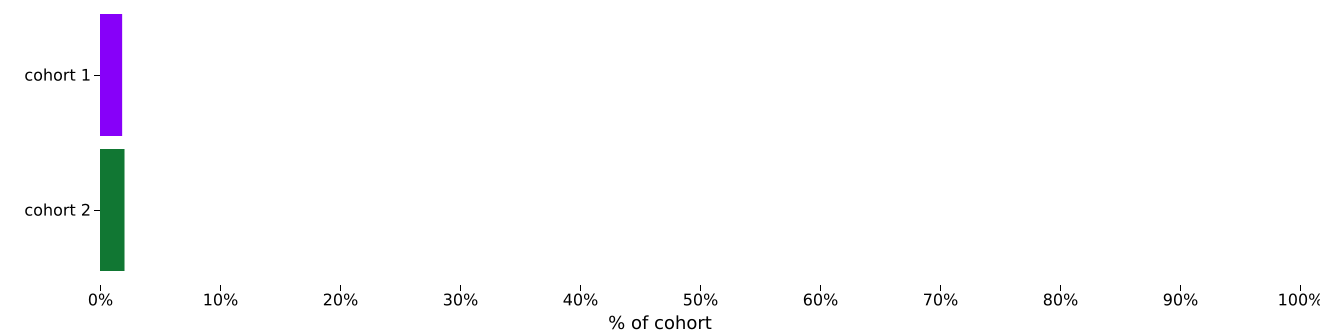 | | | | | | | |
|  | |  | | | 2,792 patients in Cohort 1 and 2,992 patients in Cohort 2 were excluded from results because they had the outcome prior to the time window. | | | | | | | |
| **5 Ventilation assist** | | | | | | | | | | | | |
|  | | **Risk analysis excluding patients with outcome prior to the time window** | | | | | | | | | | |
|  |  | | | Cohort | | | Patients in cohort | Patients with outcome | Risk | | | |
|  | | |  | 1 | | OSA + PE V4 | 72,249 | 1,623 | 0.022 | | | |
|  | | |  | 2 | | PE population w/o sleep disorder V4 | 71,935 | 1,757 | 0.024 | | | |
|  | | | | | | | | | | | | |
|  | | |  |  | | |  | 95% CI | z | p |  |  |
|  | | |  | **Risk Difference** | | | -0.002 | (-0.004, -0.000) | -2.460 | 0.014 |  |  |
|  | | |  | **Risk Ratio** | | | 0.920 | (0.860, 0.983) | N/A | N/A |  |  |
|  | | |  | **Odds Ratio** | | | 0.918 | (0.857, 0.983) | N/A | N/A |  |  |
|  | | | | | | | | | | | | |
|  | |  | | | 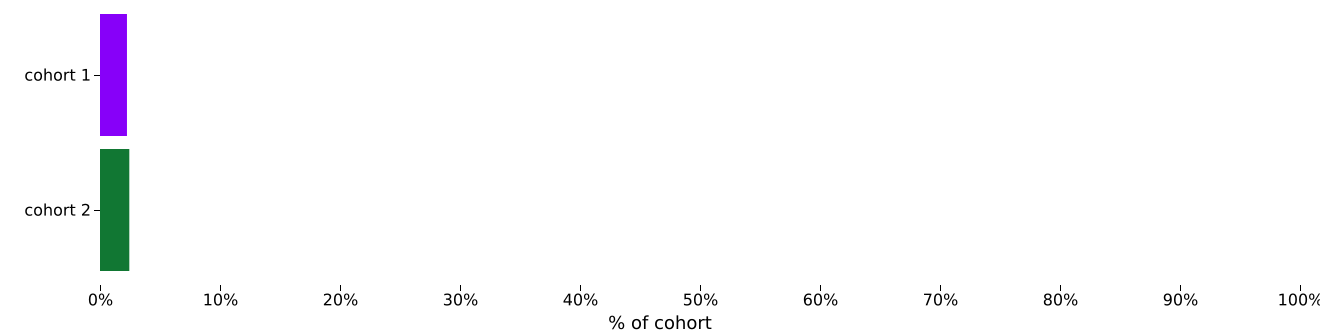 | | | | | | | |
|  | |  | | | 4,387 patients in Cohort 1 and 4,701 patients in Cohort 2 were excluded from results because they had the outcome prior to the time window. | | | | | | | |
| **6 GI bleed** | | | | | | | | | | | | |
|  | | **Risk analysis excluding patients with outcome prior to the time window** | | | | | | | | | | |
|  |  | | | Cohort | | | Patients in cohort | Patients with outcome | Risk | | | |
|  | | |  | 1 | | OSA + PE V4 | 67,749 | 741 | 0.011 | | | |
|  | | |  | 2 | | PE population w/o sleep disorder V4 | 67,931 | 997 | 0.015 | | | |
|  | | | | | | | | | | | | |
|  | | |  |  | | |  | 95% CI | z | p |  |  |
|  | | |  | **Risk Difference** | | | -0.004 | (-0.005, -0.003) | -6.124 | 0.000 |  |  |
|  | | |  | **Risk Ratio** | | | 0.745 | (0.678, 0.819) | N/A | N/A |  |  |
|  | | |  | **Odds Ratio** | | | 0.742 | (0.675, 0.817) | N/A | N/A |  |  |
|  | | | | | | | | | | | | |
|  | |  | | | 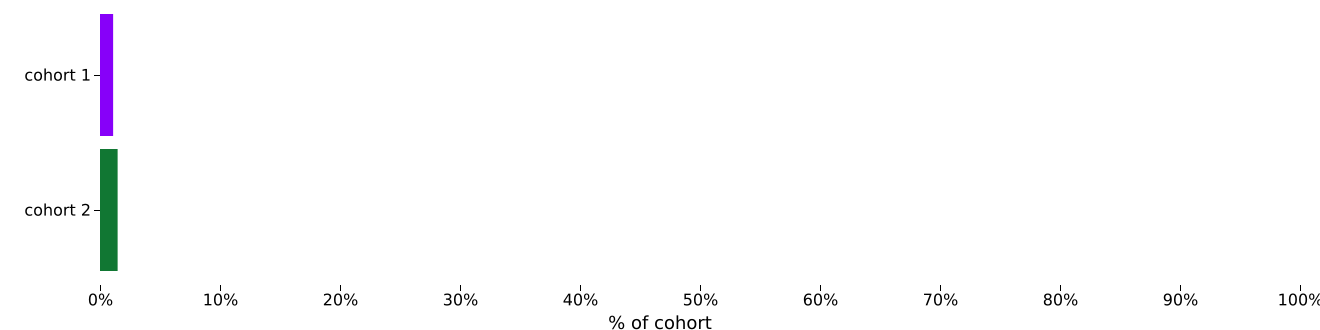 | | | | | | | |
|  | |  | | | 8,887 patients in Cohort 1 and 8,705 patients in Cohort 2 were excluded from results because they had the outcome prior to the time window. | | | | | | | |
| **7 Intracerebral bleed** | | | | | | | | | | | | |
|  | | **Risk analysis excluding patients with outcome prior to the time window** | | | | | | | | | | |
|  |  | | | Cohort | | | Patients in cohort | Patients with outcome | Risk | | | |
|  | | |  | 1 | | OSA + PE V4 | 75,250 | 144 | 0.002 | | | |
|  | | |  | 2 | | PE population w/o sleep disorder V4 | 75,057 | 233 | 0.003 | | | |
|  | | | | | | | | | | | | |
|  | | |  |  | | |  | 95% CI | z | p |  |  |
|  | | |  | **Risk Difference** | | | -0.001 | (-0.002, -0.001) | -4.614 | 0.000 |  |  |
|  | | |  | **Risk Ratio** | | | 0.616 | (0.501, 0.759) | N/A | N/A |  |  |
|  | | |  | **Odds Ratio** | | | 0.616 | (0.500, 0.758) | N/A | N/A |  |  |
|  | | | | | | | | | | | | |
|  | |  | | | 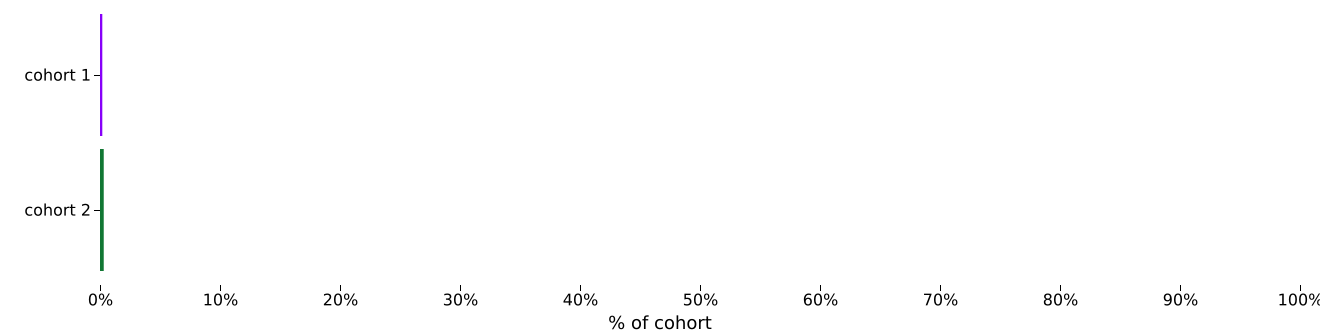 | | | | | | | |
|  | |  | | | 1,386 patients in Cohort 1 and 1,579 patients in Cohort 2 were excluded from results because they had the outcome prior to the time window. | | | | | | | |
| **8 SDH** | | | | | | | | | | | | |
|  | | **Risk analysis excluding patients with outcome prior to the time window** | | | | | | | | | | |
|  |  | | | Cohort | | | Patients in cohort | Patients with outcome | Risk | | | |
|  | | |  | 1 | | OSA + PE V4 | 75,857 | 94 | 0.001 | | | |
|  | | |  | 2 | | PE population w/o sleep disorder V4 | 75,823 | 117 | 0.002 | | | |
|  | | | | | | | | | | | | |
|  | | |  |  | | |  | 95% CI | z | p |  |  |
|  | | |  | **Risk Difference** | | | -0.000 | (-0.001, 0.000) | -1.588 | 0.112 |  |  |
|  | | |  | **Risk Ratio** | | | 0.803 | (0.612, 1.053) | N/A | N/A |  |  |
|  | | |  | **Odds Ratio** | | | 0.803 | (0.612, 1.053) | N/A | N/A |  |  |
|  | | | | | | | | | | | | |
|  | |  | | | 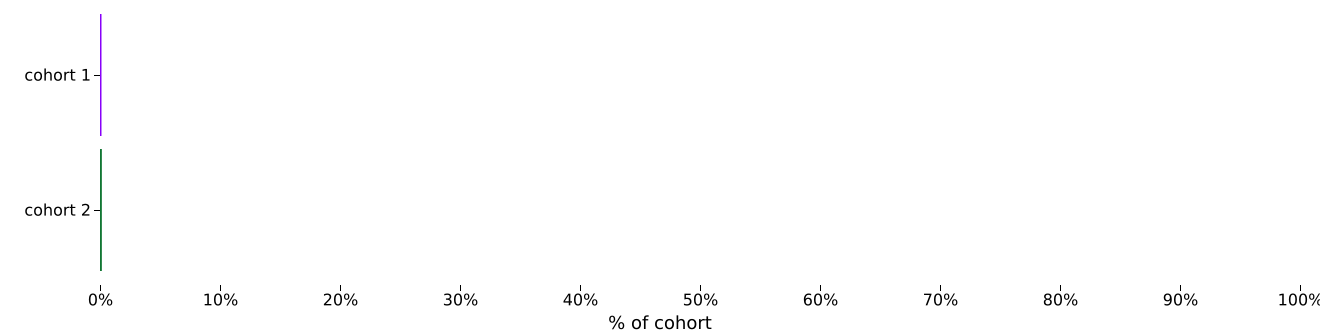 | | | | | | | |
|  | |  | | | 779 patients in Cohort 1 and 813 patients in Cohort 2 were excluded from results because they had the outcome prior to the time window. | | | | | | | |
| **9 ED** | | | | | | | | | | | | |
|  | | **Risk analysis excluding patients with outcome prior to the time window** | | | | | | | | | | |
|  |  | | | Cohort | | | Patients in cohort | Patients with outcome | Risk | | | |
|  | | |  | 1 | | OSA + PE V4 | 71,596 | 2,401 | 0.034 | | | |
|  | | |  | 2 | | PE population w/o sleep disorder V4 | 71,527 | 2,470 | 0.035 | | | |
|  | | | | | | | | | | | | |
|  | | |  |  | | |  | 95% CI | z | p |  |  |
|  | | |  | **Risk Difference** | | | -0.001 | (-0.003, 0.001) | -1.040 | 0.298 |  |  |
|  | | |  | **Risk Ratio** | | | 0.971 | (0.919, 1.026) | N/A | N/A |  |  |
|  | | |  | **Odds Ratio** | | | 0.970 | (0.916, 1.027) | N/A | N/A |  |  |
|  | | | | | | | | | | | | |
|  | |  | | | 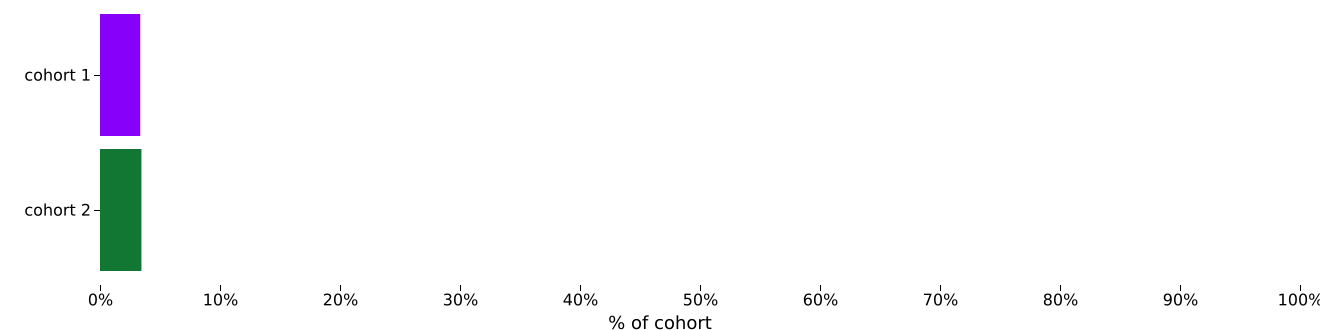 | | | | | | | |
|  | |  | | | 5,040 patients in Cohort 1 and 5,109 patients in Cohort 2 were excluded from results because they had the outcome prior to the time window. | | | | | | | |
| **10 Unnamed Outcome** | | | | | | | | | | | | |
|  | | **Risk analysis excluding patients with outcome prior to the time window** | | | | | | | | | | |
|  |  | | | Cohort | | | Patients in cohort | Patients with outcome | Risk | | | |
|  | | |  | 1 | | OSA + PE V4 | 75,778 | 737 | 0.010 | | | |
|  | | |  | 2 | | PE population w/o sleep disorder V4 | 75,934 | 766 | 0.010 | | | |
|  | | | | | | | | | | | | |
|  | | |  |  | | |  | 95% CI | z | p |  |  |
|  | | |  | **Risk Difference** | | | -0.000 | (-0.001, 0.001) | -0.712 | 0.477 |  |  |
|  | | |  | **Risk Ratio** | | | 0.964 | (0.872, 1.066) | N/A | N/A |  |  |
|  | | |  | **Odds Ratio** | | | 0.964 | (0.871, 1.067) | N/A | N/A |  |  |
|  | | | | | | | | | | | | |
|  | |  | | | 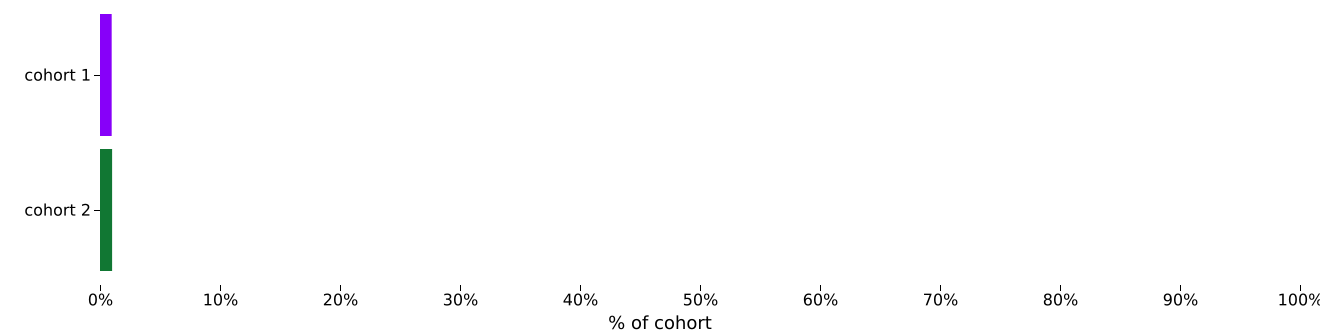 | | | | | | | |
|  | |  | | | 858 patients in Cohort 1 and 702 patients in Cohort 2 were excluded from results because they had the outcome prior to the time window. | | | | | | | |

# Appendix A – Text Representation of the Cohorts Definition

This section lists all terms used in the definitions of the two cohorts.

### Query Criteria for Cohort 1 (query name: OSA + PE V4)

Patients must have:
 Age (Age) (at least 18 years (most recent occurrence)).

All the following must be satisfied:

 Group 1A: The terms in this group occurred between Jan 1, 2013 and Jun 30, 2025
 Patients must have:
 Obstructive sleep apnea (adult) (pediatric) (UMLS:ICD10CM:G47.33).
 Group 1B: Any instance of Group 1B occurred on or after any instance of Group 1A
 Patients must have:
 Pulmonary embolism (UMLS:ICD10CM:I26).

### Query Criteria for Cohort 2 (query name: PE population w/o sleep disorder V4)

Patients must have:
 Age (Age) (at least 18 years (most recent occurrence)).

All the following must be satisfied:

 Group 1A: The terms in this group occurred between Jan 1, 2013 and Jun 30, 2025
 Patients must have:
 Pulmonary embolism (UMLS:ICD10CM:I26).
 Patients cannot have:
 Sleep disorders (UMLS:ICD10CM:G47).

# Appendix B – Text Representation of the Analysis Setup

This section contains the Index Event definition for each cohort.

The index event for Cohort 1 (query name: OSA + PE V4) is defined as the following:

All the following must be satisfied:

 Group 1A: The terms in this group occurred between Jan 1, 2013 and Jun 30, 2025
 Patients must have:
 Obstructive sleep apnea (adult) (pediatric) (UMLS:ICD10CM:G47.33).
 Group 1B: Any instance of Group 1B occurred on or after any instance of Group 1A
 Patients must have:
 Pulmonary embolism (UMLS:ICD10CM:I26).

The index event for Cohort 2 (query name: PE population w/o sleep disorder V4) is defined as the following:

All the following must be satisfied:

 Group 1A: The terms in this group occurred between Jan 1, 2013 and Jun 30, 2025
 Patients must have:
 Pulmonary embolism (UMLS:ICD10CM:I26).
 Patients cannot have:
 Sleep disorders (UMLS:ICD10CM:G47).

# Appendix C – Text Representation of the Outcomes Definition

This analysis includes the following outcomes:

Mortality
 Patients must have:
 Deceased (Deceased).

Cardiac arrest
 Patients must have:
 any of the following:
 Cardiac arrest (UMLS:ICD10CM:I46); or
 Cardiac arrest, cause unspecified (UMLS:ICD10CM:I46.9).

Critical Care services
 Patients must have:
 Critical Care Services (UMLS:CPT:1013729).

Intubation
 Patients must have:
 Intubation, endotracheal, emergency procedure (UMLS:CPT:31500).

Ventilation assist
 Patients must have:
 any of the following:
 Respiratory Ventilation, Less than 24 Consecutive Hours (UMLS:ICD10PCS:5A1935Z); or
 Respiratory Ventilation, 24-96 Consecutive Hours (UMLS:ICD10PCS:5A1945Z); or
 Respiratory Ventilation, Greater than 96 Consecutive Hours (UMLS:ICD10PCS:5A1955Z).

GI bleed
 Patients must have:
 Other diseases of digestive system (UMLS:ICD10CM:K92).

Intracerebral bleed
 Patients must have:
 Nontraumatic intracerebral hemorrhage (UMLS:ICD10CM:I61).

SDH
 Patients must have:
 Nontraumatic subdural hemorrhage (UMLS:ICD10CM:I62.0).

ED
 Patients must have:
 THROMBOLYTICS (NLM:VA:BL115).

Unnamed Outcome
 Patients must have:
 any of the following:
 Venous Mechanical Thrombectomy Procedures (UMLS:CPT:1006787); or
 Primary percutaneous transluminal mechanical thrombectomy, noncoronary, arterial or arterial bypass graft, including fluoroscopic guidance and intraprocedural pharmacological thrombolytic injection(s) (deprecated 2018) (UMLS:CPT:1006784); or
 Primary percutaneous transluminal mechanical thrombectomy, noncoronary, non-intracranial, arterial or arterial bypass graft, including fluoroscopic guidance and intraprocedural pharmacological thrombolytic injection(s) (UMLS:CPT:1027831); or
 Secondary percutaneous transluminal thrombectomy (eg, nonprimary mechanical, snare basket, suction technique), noncoronary, non-intracranial, arterial or arterial bypass graft, including fluoroscopic guidance and intraprocedural pharmacological thrombolytic injections, provided in conjunction with another percutaneous intervention other than primary mechanical thrombectomy (List separately in addition to code for primary procedure) (UMLS:CPT:37186).
