# Supplementary material for: Obstructive sleep apnea and outcomes in acute pulmonary embolism: A large-scale database study
Source: PLoS One. 2026 Mar 13;21(3):e0342850. doi: 10.1371/journal.pone.0342850 (PMC12987454; doi:10.1371/journal.pone.0342850)
Supplement: Attachment — Submitted filename: PONE_Reviewers response_Sharma_Nov2.docx [file pone.0342850.s003.docx]

PONE-D-25-51061

Obstructive sleep apnea and outcomes in acute pulmonary embolism: a large-scale database study

PLOS ONE

Dear Dr. Sharma,

Thank you for submitting your manuscript to PLOS ONE. After careful consideration, we feel that it has merit but does not fully meet PLOS ONE’s publication criteria as it currently stands. Therefore, we invite you to submit a revised version of the manuscript that addresses the points raised during the review process.

Please submit your revised manuscript by  Dec 06 2025 11:59PM. If you will need more time than this to complete your revisions, please reply to this message or contact the journal office at [plosone@plos.org](mailto:plosone@plos.org). When you're ready to submit your revision, log on to https://www.editorialmanager.com/pone/ and select the 'Submissions Needing Revision' folder to locate your manuscript file.

Please include the following items when submitting your revised manuscript:

- A rebuttal letter that responds to each point raised by the academic editor and reviewer(s). You should upload this letter as a separate file labeled 'Response to Reviewers'.
- A marked-up copy of your manuscript that highlights changes made to the original version. You should upload this as a separate file labeled 'Revised Manuscript with Track Changes'.
- An unmarked version of your revised paper without tracked changes. You should upload this as a separate file labeled 'Manuscript'.

If you would like to make changes to your financial disclosure, please include your updated statement in your cover letter. Guidelines for resubmitting your figure files are available below the reviewer comments at the end of this letter.

If applicable, we recommend that you deposit your laboratory protocols in protocols.io to enhance the reproducibility of your results. Protocols.io assigns your protocol its own identifier (DOI) so that it can be cited independently in the future. For instructions see: [https://journals.plos.org/plosone/s/submission-guidelines#loc-laboratory-protocols](https://nam04.safelinks.protection.outlook.com/?url=https%3A%2F%2Ftrack.editorialmanager.com%2FCL0%2Fhttps%3A%252F%252Fjournals.plos.org%252Fplosone%252Fs%252Fsubmission-guidelines%2523loc-laboratory-protocols%2F1%2F010f019a0f15220a-c2c1e601-b4ec-44bb-8792-5580eb0af8d7-000000%2FqSyQebuF1Kk_ki3YsEH6gmVag4CNn8Clz6O8Bi6LFk4%3D234&data=05%7C02%7Csunil.sharma%40hsc.wvu.edu%7Cf5d80f1779264a92ca4308de11e321e9%7Ca2d1f95f851044248ae15c596bdbd578%7C0%7C0%7C638967864704305343%7CUnknown%7CTWFpbGZsb3d8eyJFbXB0eU1hcGkiOnRydWUsIlYiOiIwLjAuMDAwMCIsIlAiOiJXaW4zMiIsIkFOIjoiTWFpbCIsIldUIjoyfQ%3D%3D%7C0%7C%7C%7C&sdata=J8JJImFVSyxrHFrAOrQtjInLo5pyb5Ae0I3KExEHKVk%3D&reserved=0). Additionally, PLOS ONE offers an option for publishing peer-reviewed Lab Protocol articles, which describe protocols hosted on protocols.io. Read more information on sharing protocols at [https://plos.org/protocols?utm_medium=editorial-email&utm_source=authorletters&utm_campaign=protocols](https://nam04.safelinks.protection.outlook.com/?url=https%3A%2F%2Ftrack.editorialmanager.com%2FCL0%2Fhttps%3A%252F%252Fplos.org%252Fprotocols%253Futm_medium%3Deditorial-email%2526utm_source%3Dauthorletters%2526utm_campaign%3Dprotocols%2F1%2F010f019a0f15220a-c2c1e601-b4ec-44bb-8792-5580eb0af8d7-000000%2FE3FDo7V1WRjXUi8skU-Cya8kkZKNBYVEfV4rNzmYFTg%3D234&data=05%7C02%7Csunil.sharma%40hsc.wvu.edu%7Cf5d80f1779264a92ca4308de11e321e9%7Ca2d1f95f851044248ae15c596bdbd578%7C0%7C0%7C638967864704353648%7CUnknown%7CTWFpbGZsb3d8eyJFbXB0eU1hcGkiOnRydWUsIlYiOiIwLjAuMDAwMCIsIlAiOiJXaW4zMiIsIkFOIjoiTWFpbCIsIldUIjoyfQ%3D%3D%7C0%7C%7C%7C&sdata=EL%2Bz%2FSHbN%2B0IsNLYgcdep7Oci9inG3NmmEd33AR%2BpD0%3D&reserved=0).

We look forward to receiving your revised manuscript.

Kind regards,

Kartikeya Rajdev, MD
Academic Editor
PLOS ONE

Journal Requirements:

When submitting your revision, we need you to address these additional requirements.

1. Please ensure that your manuscript meets PLOS ONE's style requirements, including those for file naming. The PLOS ONE style templates can be found at

<https://journals.plos.org/plosone/s/file?id=wjVg/PLOSOne_formatting_sample_main_body.pdf> and

[https://journals.plos.org/plosone/s/file?id=ba62/PLOSOne_formatting_sample_title_authors_affiliations.pdf](https://nam04.safelinks.protection.outlook.com/?url=https%3A%2F%2Ftrack.editorialmanager.com%2FCL0%2Fhttps%3A%252F%252Fjournals.plos.org%252Fplosone%252Fs%252Ffile%253Fid%3Dba62%252FPLOSOne_formatting_sample_title_authors_affiliations.pdf%2F1%2F010f019a0f15220a-c2c1e601-b4ec-44bb-8792-5580eb0af8d7-000000%2FbniwbiaU7pfjmVc7uTkUxlGihZlvi091Poh0ZqK-GC0%3D234&data=05%7C02%7Csunil.sharma%40hsc.wvu.edu%7Cf5d80f1779264a92ca4308de11e321e9%7Ca2d1f95f851044248ae15c596bdbd578%7C0%7C0%7C638967864704380722%7CUnknown%7CTWFpbGZsb3d8eyJFbXB0eU1hcGkiOnRydWUsIlYiOiIwLjAuMDAwMCIsIlAiOiJXaW4zMiIsIkFOIjoiTWFpbCIsIldUIjoyfQ%3D%3D%7C0%7C%7C%7C&sdata=HFvn9%2FIi5Md522HGYzy4OpIq%2FUx56hOBGMAAQ%2FKr5M8%3D&reserved=0)

2. Thank you for stating the following in the Competing Interests section:
“Dr. Sharma declares receives grant and support for attending meetings or travel from West Virginia University (WVU) and National Science Foundation (NSF); SS has been on the speakers bureau of Zoll Respicardia Inc.(Until Nov 2024). SS has received Grant funding from INARI Medical Inc PEERLESS II trial, NIH RECOVER trial, rEST trial by Zoll Respicardia Inc.; and holds patents with WVU (invention numbers: US2023/0122156) that has been licensed by Premash Inc. SS is consultant for Premash Inc.

Dr. Alawad, Dr. Al-Saeed and Dr. Jarrar have no conflicts to declare”

Please confirm that this does not alter your adherence to all PLOS ONE policies on sharing data and materials, by including the following statement: **"This does not alter our adherence to PLOS ONE policies on sharing data and materials.”** (as detailed online in our guide for authors <http://journals.plos.org/plosone/s/competing-interests>). If there are restrictions on sharing of data and/or materials, please state these. Please note that we cannot proceed with consideration of your article until this information has been declared.

**Response: Confirmed and included**

Please include your updated Competing Interests statement in your cover letter; we will change the online submission form on your behalf.

**Response: Done**

3. Thank you for uploading your study's underlying data set. Unfortunately, the repository you have noted in your Data Availability statement does not qualify as an acceptable data repository according to PLOS's standards.

At this time, please upload the **minimal data set necessary to replicate your study's findings to a stable, public repository (such as figshare or Dryad) and provide us with the relevant URLs, DOIs, or accession numbers that may be used to access these data**. For a list of recommended repositories and additional information on PLOS standards for data deposition, please see [https://journals.plos.org/plosone/s/recommended-repositories](https://nam04.safelinks.protection.outlook.com/?url=https%3A%2F%2Ftrack.editorialmanager.com%2FCL0%2Fhttps%3A%252F%252Fjournals.plos.org%252Fplosone%252Fs%252Frecommended-repositories%2F1%2F010f019a0f15220a-c2c1e601-b4ec-44bb-8792-5580eb0af8d7-000000%2FWgcb6lEVVVqE27_rAmUQXxYdF-MD9Sz1343qSbR4P7M%3D234&data=05%7C02%7Csunil.sharma%40hsc.wvu.edu%7Cf5d80f1779264a92ca4308de11e321e9%7Ca2d1f95f851044248ae15c596bdbd578%7C0%7C0%7C638967864704400372%7CUnknown%7CTWFpbGZsb3d8eyJFbXB0eU1hcGkiOnRydWUsIlYiOiIwLjAuMDAwMCIsIlAiOiJXaW4zMiIsIkFOIjoiTWFpbCIsIldUIjoyfQ%3D%3D%7C0%7C%7C%7C&sdata=pfCkUaRm5%2FXBcCaeiVRQqaiVwT3IVCn84qqqmPOj4mo%3D&reserved=0).

**Response: The data used in this study were accessed through the TriNetX Research Network, a global federated health research platform. The data comprise aggregated, de-identified patient information that cannot be shared or downloaded in raw form per the data use agreement and network governance policies.**

**Researchers interested in replicating or validating these findings can request access to TriNetX through institutional membership or by contacting TriNetX (**[**https://www.trinetx.com**](https://www.trinetx.com)**) to obtain access to the same analytic tools and datasets** under a similar data use agreement. As per TriNetX policy and data use agreements, **the underlying patient-level data cannot be shared or deposited in a public repository**. However, all analyses were conducted within the TriNetX platform using reproducible query criteria, which are attached as a supplement.

4. We notice that your **supplementary [figures/tables] are included in the manuscript file. Please remove them and upload them with the file type 'Supporting Information'.** Please ensure that each Supporting Information file has a legend listed in the manuscript after the references list.

**Done**

5. If the reviewer comments include a recommendation to cite specific previously published works, please review and evaluate these publications to determine whether they are relevant and should be cited. There is **no requirement to cite these works unless** the editor has indicated otherwise. 

**Noted. None was added**

[Note: HTML markup is below. Please do not edit.]

Reviewers' comments:

Reviewer's Responses to Questions

**Comments to the Author**

1. Is the manuscript technically sound, and do the data support the conclusions?

The manuscript must describe a technically sound piece of scientific research with data that supports the conclusions. Experiments must have been conducted rigorously, with appropriate controls, replication, and sample sizes. The conclusions must be drawn appropriately based on the data presented.

Reviewer #1: Yes

Reviewer #2: Yes

Reviewer #3: Yes

Reviewer #4: Partly

2. Has the statistical analysis been performed appropriately and rigorously?

Reviewer #1: Yes

Reviewer #2: Yes

Reviewer #3: Yes

Reviewer #4: Yes

3. Have the authors made all data underlying the findings in their manuscript fully available?

The [PLOS Data policy](https://nam04.safelinks.protection.outlook.com/?url=http%3A%2F%2Ftrack.editorialmanager.com%2FCL0%2Fhttp%3A%252F%252Fwww.plosone.org%252Fstatic%252Fpolicies.action%2523sharing%2F1%2F010f019a0f15220a-c2c1e601-b4ec-44bb-8792-5580eb0af8d7-000000%2F25TU35cPk3T1JF2J7I7C23g7NyWcLrtAUw5VR395v0I%3D234&data=05%7C02%7Csunil.sharma%40hsc.wvu.edu%7Cf5d80f1779264a92ca4308de11e321e9%7Ca2d1f95f851044248ae15c596bdbd578%7C0%7C0%7C638967864704418802%7CUnknown%7CTWFpbGZsb3d8eyJFbXB0eU1hcGkiOnRydWUsIlYiOiIwLjAuMDAwMCIsIlAiOiJXaW4zMiIsIkFOIjoiTWFpbCIsIldUIjoyfQ%3D%3D%7C0%7C%7C%7C&sdata=EvPIEtiz5iiNDCm8%2Fak1HkFEa1%2BaKg3nLS5WWwBbfRg%3D&reserved=0) requires authors to make all data underlying the findings described in their manuscript fully available without restriction, with rare exception (please refer to the Data Availability Statement in the manuscript PDF file). The data should be provided as part of the manuscript or its supporting information, or deposited to a public repository. For example, in addition to summary statistics, the data points behind means, medians and variance measures should be available. If there are restrictions on publicly sharing data—e.g. participant privacy or use of data from a third party—those must be specified.

Reviewer #1: Yes

Reviewer #2: Yes

Reviewer #3: Yes

Reviewer #4: Yes

4. Is the manuscript presented in an intelligible fashion and written in standard English?

PLOS ONE does not copyedit accepted manuscripts, so the language in submitted articles must be clear, correct, and unambiguous. Any typographical or grammatical errors should be corrected at revision, so please note any specific errors here.

Reviewer #1: Yes

Reviewer #2: Yes

Reviewer #3: Yes

Reviewer #4: Yes

5. Review Comments to the Author

Please use the space provided to explain your answers to the questions above. You may also include additional comments for the author, including concerns about dual publication, research ethics, or publication ethics. (Please upload your review as an attachment if it exceeds 20,000 characters)

Reviewer #1: No OSA severity (AHI index) or PE severity (massive/submassive/subsegmental) available. This limitation should be explicitly emphasized in the Major Limitations since severity likely affects mortality

**Response: We thank the reviewer for the comments. We have already mentioned OSA severity and PE severity in the limitations section. As per the suggestions we have added (massive/ submassive/ subsegmental) to the sentence)**

Absence of CPAP/PAP adherence **information weakens mechanistic interpretation this should be major not a minor limitation.**

**Response: We believe we have adequately stressed the limitation of lack of CPAP adherence data- “ Furthermore, In addition, while our findings indicate a potentially "protective" relationship, we caution against reading too much into this observation due to the nature of our study and missing key data about important factors such as CPAP treatment”**

OSA appears to positively influence against 30-day mortality- consider revising to association language (“**OSA was associated with lower observed 30-day mortality**)

**Response :We appreciate the reviewer comments and agree with the above suggestion. We have revised the language as recommended.**

Reviewer #2: The study conclusion is some what paradoxical it does add some valuable information in terms of complex interplay between OSA and PE. The authors rightly acknowledge the strengths and limitations especially OSA severity, PE severity and anticoagulation regimens. Also since the **study period is 2013-2024 where there is significant increase in usage of DOACs there is no information about this in the study.**

**Response: We than the review for their astute observation. We however did include DOAC’s in the analysis. This is mentioned now more explicitly in the methods section.**

Reviewer #3: I enjoyed reviewing this manuscript. Despite study limitations, as indicated in the manuscript, this study provides some valuable piece of information about the complex relationship between OSA and PE. It would have really been helpful to know the relationship between PAP compliance and PE**.**

**Response: We are glad that the reviewer enjoyed the manuscript. We agree that the study provides important insight into the relationship between PAP and PE. Unfortunately the PAP compliance information is not included in the TriNetX database, otherwise we would have gladly included that data.**

Reviewer #4: This manuscript presents a large, contemporary TriNetX-based analysis evaluating how obstructive sleep apnea (OSA) influences the risk and short-term outcomes of acute pulmonary embolism (PE). The topic is timely and clinically relevant. The study adds novelty by including both incidence data and 30-day post-PE outcomes, particularly cardiac arrest, which extends prior inpatient-only reports such as Joshi et al., Pulmonary Circulation 2021. The dataset spans 2013–2024, reflecting modern therapeutic practices including DOACs and catheter-directed interventions. However, several methodological and reporting issues limit interpretability. There are internal inconsistencies in sample sizes between the Abstract and Results, residual BMI imbalance after propensity matching, unclear encounter definitions (hospitalized vs outpatient PE), and incomplete details on mortality ascertainment. The question is important and the data are strong, but the paper requires a major revision before it can be considered further.

**Response: We thank the reviewers for their comments. We have rechecked and there are no internal inconsistencies between abstract and the results. We do acknowledge that the data required more explanation i.e. clearly stating that outcome data was based on propensity matching and not overall sample size. This has been clarified in the abstract.**

**Response: The residual BMI imbalance has been addressed in the limitations section. As noted, although a difference in BMI persisted despite propensity matching, both groups remained within the same obesity class. This has been explicitly acknowledged in the limitations.**

**By default, acute pulmonary embolism is typically managed in the inpatient setting. However, it is possible that a subset of patients in the sample may have been treated in ambulatory settings. Because the ICD code is the same for both, the dataset does not allow differentiation between inpatient and outpatient management. This clarification has also been incorporated into the limitations section. Mortality data was limited to all-cause mortality. Mortality language has also been changed as suggested from “ positively influencing” to “OSA was associated with lower observed 30-day mortality”**

6. PLOS authors have the option to publish the peer review history of their article ([what does this mean?](https://nam04.safelinks.protection.outlook.com/?url=https%3A%2F%2Ftrack.editorialmanager.com%2FCL0%2Fhttps%3A%252F%252Fjournals.plos.org%252Fplosone%252Fs%252Feditorial-and-peer-review-process%2523loc-peer-review-history%2F1%2F010f019a0f15220a-c2c1e601-b4ec-44bb-8792-5580eb0af8d7-000000%2FI-36fvn6O1dNrWvPMWS96_jRqPxUdLO8Y-3UdeMlDL0%3D234&data=05%7C02%7Csunil.sharma%40hsc.wvu.edu%7Cf5d80f1779264a92ca4308de11e321e9%7Ca2d1f95f851044248ae15c596bdbd578%7C0%7C0%7C638967864704441910%7CUnknown%7CTWFpbGZsb3d8eyJFbXB0eU1hcGkiOnRydWUsIlYiOiIwLjAuMDAwMCIsIlAiOiJXaW4zMiIsIkFOIjoiTWFpbCIsIldUIjoyfQ%3D%3D%7C0%7C%7C%7C&sdata=J%2F%2BVbZA3GebN1f8hAu5kqJ5QN4ImfY70%2F%2BGN3OuAFWk%3D&reserved=0)). If published, this will include your full peer review and any attached files.


If you choose “no”, your identity will remain anonymous but your review may still be made publi
