# Supplementary material for: Obstructive sleep apnea and outcomes in acute pulmonary embolism: A large-scale database study
Source: PLoS One. 2026 Mar 13;21(3):e0342850. doi: 10.1371/journal.pone.0342850 (PMC12987454; doi:10.1371/journal.pone.0342850)
Supplement: Attachment — Submitted filename: Review Comments to the Author_response_Dec15.docx [file pone.0342850.s004.docx]

Response to review Comments to the Author

Please use the space provided to explain your answers to the questions above. You may also include additional comments for the author, including concerns about dual publication, research ethics, or publication ethics. (Please upload your review as an attachment if it exceeds 20,000 characters)

Reviewer #1: It is well written paper however the limitation are pertinent to the type of data you are extracting. I have noticed the suggestion revisions are appropriately addressed

**We appreciate the reviewers comments**

Reviewer #2: lack of inpatient vs outpatient differentiation of PE as ICD codes frequently capture chronic PE, follow up evaluation in the out patient setting.
Also with out PE severity stratification it is some what premature to assume that there is physiological protection from obesity.

**Finally, as the study relied on ICD and procedure codes, its conclusions depend on consistent and accurate physician coding. Coding bias may be introduced if coding practices vary across healthcare organizations, including differences in how conditions are classified or managed in inpatient versus outpatient settings.**

**Furthermore, In addition, while our findings indicate a potentially "protective" relationship, we caution against reading too much into this observation due to the nature of our study and missing key data about important factors such as CPAP treatment.**

Reviewer #3: (No Response)

Reviewer #4: Thank you for the revised submission and for the thoughtful responses to the reviewer comments. The authors have adequately addressed the previously raised concerns, including clarification of cohort definitions, refinement of associative language, expansion of the limitations section to appropriately acknowledge unavailable data on OSA severity, pulmonary embolism severity, and PAP adherence, and clarification regarding anticoagulation strategies, mortality ascertainment, and ICD-based encounter definitions. The revisions have improved the clarity, transparency, and interpretability of the manuscript without altering its core findings

**We appreciate the reviewers comments**
